# Achievements and challenges of implementation in a mature iCCM programme: Malawi case study

**DOI:** 10.7189/jogh.09.010807

**Published:** 2019-06

**Authors:** Kirsten Zalisk, Tanya Guenther, Debra Prosnitz, Humphreys Nsona, Emmanuel Chimbalanga, Salim Sadruddin

**Affiliations:** 1 ICF, Rockville, Maryland, USA; 2 Abt Associates Inc, Dili, Timor-Leste; 3 Republic of Malawi Ministry of Health, Lilongwe, Malawi; 4 Management Sciences for Health, Lilongwe, Malawi; 5 World Health Organization, Geneva, Switzerland

## Abstract

**Background:**

Malawi has a mature integrated community case management (iCCM) programme that is led by the Ministry of Health (MOH) but that still relies on donor support. From 2013 until 2017, under the Rapid Access Expansion (RAcE) programme, the World Health Organization supported the MOH to expand and strengthen iCCM services in four districts. This paper examines Malawi’s iCCM programme performance and implementation strength in RAcE districts to further strengthen the broader programme.

**Methods:**

Baseline and endline household surveys were conducted in iCCM-eligible areas of RAcE districts. Primary caregivers of recently-sick children under five were interviewed to assess changes in care-seeking and treatment over the project period. Health surveillance assistants (HSAs) were surveyed at endline to assess iCCM implementation strength.

**Results:**

Care-seeking from HSAs and treatment of fever improved over the project period. At endline, however, less than half of sick children were brought to an HSA, many caregivers reported a preference for providers other than HSAs, and perceptions of HSAs as trusted providers of high-quality, convenient care had decreased. HSA supervision and mentorship were below MOH targets. Stockouts of malaria medicines were associated with decreased care-seeking from HSAs. Thirty percent of clusters had limited or no access to iCCM (no HSA or an HSA providing iCCM services less than 2 days per week); 50% had moderate access (an HSA providing iCCM services 2 to 4 days per week; and 20% had high access (a resident HSA providing iCCM services 5 or more days per week). Moderate access to iCCM was associated with increased care-seeking from HSAs, increased treatment by HSAs, and more positive perceptions of HSAs compared to areas with limited or no access. Areas with high access to iCCM did not show further improvements above areas with moderate access.

**Conclusions:**

Availability of well-equipped and supported HSAs is critical to the provision of iCCM services. Additional qualitative research is needed to examine challenges and to inform potential solutions. Malawi’s mature iCCM programme has a strong foundation but can be improved to strengthen the continuity of care from communities to facilities and to ultimately improve child health outcomes.

Community health workers (CHWs), referred to as health surveillance assistants (HSAs), have been working in Malawi since the 1960s [1]. HSAs initially focused on health prevention and promotion activities, but in 2008, they began to take on curative activities when Malawi adopted integrated community case management (iCCM) and expanded the HSA workforce to help reach the country’s goal of universal coverage of key child health interventions [1].

Integrated community case management (iCCM) is an equity-focused strategy that can improve access to and availability of essential treatment services for children by training, equipping, and supporting CHWs to manage – assess, classify, and treat – cases of malaria, pneumonia, and diarrhea among children under five years of age at the community level [2]. Communities farther than eight kilometers from a health facility or with difficult access because of geographical terrain or natural barriers were designated as being located in hard-to-reach areas (HTRAs) in Malawi and thus eligible for iCCM. District health management teams identified 3452 HTRAs [1], and iCCM rollout began in 2009 [3]. By September 2011, 2709 HTRAs had iCCM services [1]. Throughout its implementation, the iCCM programme in Malawi has benefited from the financial support received from global funding agencies and technical and logistical support from non-governmental organizations, primarily at the district or sub-district levels [4].

HSAs are centrally recruited and on the payroll of the MOH, but they are deployed to and stationed in the communities that they serve [5], providing iCCM services from their village clinics [6] in addition to providing other promotion and prevention activities at the community and health facility levels. HSAs are meant to serve approximately 1000 residents, but in practice they often have catchment areas of 2000 residents or more [5]. HSAs have at least 2 years of secondary school education and receive 10 weeks of pre-service training [1]. HSAs implementing iCCM complete six additional days of focused iCCM training [1] and are provided a drug box containing iCCM supplies. These supplies are replenished monthly at each HSA’s supervisory health facility, where HSAs also submit their reporting forms and go for mentoring sessions with clinicians.

Responsibilities of HSAs providing iCCM services include assessing, classifying, and treating children ages 2-59 months who present with common childhood illnesses – fever, cough, fast or difficult breathing, diarrhea, and eye infections. HSAs also screen for malnutrition and identify signs of severe illnesses and refer those children, and other children with illness that they cannot treat, to a health facility. In addition to iCCM-related activities, HSAs are responsible for conducting health promotion, prevention, supervisory, surveillance, and assessment activities [1,7]. Because they are responsible for implementing numerous activities in their communities and at health facilities, they cannot provide iCCM services every day. At a minimum, HSAs are supposed to open their village clinics two days per week and provide iCCM services on demand outside of their village clinic hours [4].

The iCCM programme in Malawi is a mature one – having been rolled out almost a decade ago and scaled up to all districts. In 2013-2014, an independent national-level evaluation was conducted to assess the extent to which iCCM was associated with increases in care-seeking for childhood illness and accelerated declines in under-five mortality [8]. The evaluation showed that iCCM did not lead to an increase in care-seeking for childhood illness or impact under-five mortality, but it did show an increase in care-seeking from HSAs between 2010 and 2014 [8]. The evaluation further showed that iCCM implementation needed further strengthening. Among the 3717 surveyed HSAs trained in iCCM, 91% were providing iCCM services, but only 70% of those providing iCCM services lived in their catchment areas. Furthermore, during the three months prior to the assessment, 57% had key iCCM medicines in stock; 44% were supervised; and 58% received clinical mentorship, though results varied widely among the 27 districts included in the evaluation [4].

From 2013 until 2017, under the Rapid Access Expansion (RAcE) programme, the World Health Organization supported the MOH to expand and strengthen iCCM services in four districts: Dedza, Mzimba North, Ntchisi, and Ntcheu. When RAcE started, iCCM was well-established across Malawi, and more than 400 HSAs trained in iCCM were already working in the four project districts. The goal of RAcE Malawi programme, therefore, was to expand and strengthen iCCM by: (1) extending services to additional hard-to-reach areas, (2) supporting the shift in iCCM-eligible areas from areas more than 8 km to areas more than 5 km from a health facility, (3) introducing malaria rapid diagnostic tests (mRDTs) at the community level, and (4) updating the first-line antibiotic that HSAs use to treat children with pneumonia. RAcE also aimed to strengthen iCCM implementation by ensuring regular supply of iCCM medicines and other commodities, strengthening HSA supervision and mentorship, and increasing community awareness of and demand for iCCM services.

The purpose of this study is to examine iCCM implementation strength and programme performance in RAcE districts to further strengthen Malawi’s mature iCCM programme.

## METHODS

### Study design

Two cross-sectional household surveys – the first at project baseline and the second at project endline – were conducted in HTRAs of the RAcE districts. The surveys used a 60×15 multi-stage cluster sampling methodology and targeted primary caregivers of children aged 2-59 months who had been sick with diarrhea, fever, or cough with difficult or fast breathing in the two weeks preceding the survey. The data were analyzed to assess changes in care-seeking and treatment over the project period. A survey of HSAs was conducted concurrently with the endline household survey to assess iCCM implementation strength. The objective of the HSA survey was to gain a better understanding of HSA background characteristics, activity levels, and support that they received to help interpret the household survey results. The baseline survey fieldwork was conducted in September 2013, and the endline survey fieldwork was conducted in August 2016.

### Study area

The target population comprised the entire RAcE project area, iCCM-eligible areas more than 5 km from a health facility, of the four project districts. The primary sampling units were 2008 national census enumerations areas (EAs). All EAs located within 5 km of a health facility were excluded from the sampling frame before 60 EAs were randomly selected proportional to population size. The same EAs, or clusters, were included in both the baseline and endline surveys.

Within each cluster, all households were listed and those without children under five who had been sick with diarrhea, fever, or cough with difficult or fast breathing in the two weeks preceding the survey were removed from the list. The survey team then randomly selected 15 households from the list to visit and 15 back-up households. The survey team visited each selected household in the first group of 15 to obtain at least 5 interviews for each illness module – diarrhea, fever, and fast breathing – for a total of at least 15 interviews per cluster, or 300 interviews per illness across the project area. If the survey team did not obtain all necessary interviews, the survey team visited back-up households to fulfill the requirements for the cluster.

At each household, if there was an eligible child, the interviewer administered the questionnaire, including all applicable illness modules, to the caregiver of the eligible child. If multiple children were eligible, and they were sick with different illnesses, their caregiver was asked about each instance of illness. If multiple children in the household were eligible for the same illness, the interviewer randomly selected one of them and interviewed his or her caregiver. If there were multiple children selected for inclusion in the survey, and the children had different primary caregivers, each primary caregiver of the selected children was interviewed.

HSAs providing iCCM services in the 60 clusters selected for the household survey comprised the HSA survey target population. The clusters did not align perfectly with HSA catchment areas; therefore, if more than one HSA was providing iCCM services in a cluster, one eligible HSA was randomly selected for interview. If the survey team did not find an HSA eligible for interview in a cluster, they recorded the reason an iCCM-trained HSA was not available.

### Survey questionnaire

The household survey questionnaire included seven modules: caregiver and household background information; caregivers’ knowledge of iCCM activities in their community; caregivers’ knowledge of childhood illness danger signs; household decision-making; and a module for each major childhood illness: fever, diarrhea, and fast breathing. The baseline questionnaire was also used at endline, but two questions were added to each illness module to gather information about reasons caregivers did not seek care at all or did not seek care from an HSA. The questionnaire was translated into the national language, Chichewa, and pretested during the enumerator and supervisor training. The survey took approximately one hour to administer in each household.

The HSA survey questionnaire contained eight sections, four of which were relevant to this study: HSA background, supervision, medicine and supplies, and iCCM activities and register review. The questionnaire was translated to Chichewa and pretested alongside the household survey questionnaire. The survey took approximately 30 minutes to administer to each HSA.

### Data collection and analysis

Both household surveys and the HSA survey were conducted using paper questionnaires. All data were double-entered into CSPro (US Census Bureau, Suitland, Maryland, USA), cleaned, and imported into Stata version 14 (StataCorp LLC, College Station, Texas, USA) for analysis. The analyst further cleaned and coded the Stata data files for analysis and calculated point estimates and 95 percent confidence intervals for background, programme performance, and implementation strength indicators. Household survey indicators were calculated for baseline and endline, accounting for cluster effects. To test for statistically significant changes in indicators between baseline and endline, the analyst used a Pearson χ^2^ test; all outcomes of interest were binary or categorical variables. Changes with p-values less than 0.05 were deemed statistically significant.

The household and HSA survey data were combined to also explore the relationship between iCCM access and caregiver perceptions of HSAs, care-seeking, and treatment coverage existed at endline. We classified the survey clusters as having limited or no access, moderate access, or high access to iCCM as follows: clusters with limited or no access did not have an HSA providing iCCM services or had an HSA who did not meet the MOH definition of functional (ie, managed at least one sick child case in past month and operated a village clinic at least two days per week); clusters with moderate access had an HSA who met the MOH definition of functional; and clusters with high access had an HSA who managed at least one sick child case in past month, operated a village clinic at least five days per week, and resided in catchment area.

## RESULTS

### Description of the sample

There were 1260 total cases of illness among 807 sick children in the baseline survey, and 1447 total cases of illness among 873 sick children in the endline survey (**Table 1**). There were 720 primary caregivers of sick children interviewed at baseline, and 783 primary caregivers of sick children interviewed at endline. The characteristics of the sick children and caregivers included in the baseline and endline surveys were similar, but at endline more children had fever in the two weeks preceding the survey (70%) compared to baseline (60%), and a larger percentage of caregivers were married at baseline (85%) compared to endline (75%).

**Table 1:** Characteristics of sick children and their caregivers included in the surveys

Child characteristic		Baseline % (95% CI)	Endline % (95% CI)		*P*-value
**Sex of sick children included in survey:**					
Male, %		51.4 (47.7-55.2)	49.8 (46.7-52.9)		0.491
Female, %		48.6 (44.8-52.3)	50.2 (47.1-53.3)		
**Age (months) of sick children included in survey:**					
2-11, %		22.1 (19.1-25.7)	22.1 (19.5-25.0)		0.724
12-23, %		24.7 (21.7-27.8)	24.6 (22.1-27.3)		
24-35, %		22.7 (19.9-25.7)	20.3 (17.5-23.4)		
36-47, %		17.2 (14.9-19.9)	20.3 (17.5-23.4)		
48-59, %		13.3 (10.7-16.3)	14.8 (12.6-17.3)		
**Two-week history of illness of sick children included in survey:**					
Had fever, %		59.9 (56.4-63.2)	70.7 (66.9-74.2)		**<0.001**
Had diarrhea, %		46.5 (43.7-49.2)	46.1 (43.3-48.9)		0.811
Had cough with difficult or fast breathing, %		58.5 (55.4-61.6)	60.0 (56.6-63.3)		0.461
Average number of illnesses, N		1.6	1.8		
**Total number of sick children included in survey**		**807**	**873**		
**Cases of illness among sick children included in survey:**					
Fever, N		455	571		
Diarrhea, N		364	387		
Cough with difficult or fast breathing, N		441	489		
**Total number of sick child cases included in survey**		**1260**	**1447**		
**Caregiver characteristic:**					
**Age (years):**					
15-24	35.8 (32.7-39.1)			40.2 (36.4-44.2)	0.306
25-34	44.3 (40.6-48.1)			40.0 (36.7-43.4)	
35-44	15.6 (13.3-18.1)			15.7 (13.2-18.6)	
45-76	4.3 (2.9-6.4)			4.1 (2.7-6.2)	
Mean age (years)	29			28	
**Highest level of education:**					
None	14.4 (11.1-18.6)			12.1 (9.5-15.4)	0.063
Primary,≤4 years	32.2 (28.4-36.3)			36.0 (31.8-40.5)	
Primary,≥5 year	43.5 (38.6-48.5)			39.1 (34.3-44.1)	
Secondary or higher	9.9 (7.6-12.7)			12.8 (10.2-15.8)	
**Marital status:**					
Currently married	84.9 (81.1-88.0)			75.4 (71.4-78.9)	**<0.001**
Not married but living with partner	3.8 (2.2-6.4)			9.2 (6.4-13.1)	
Not in union	11.4 (8.9-14.5)			15.5 (13.1-18.1)	
**Total number of caregivers**	**720**			**783**	

On average, caregivers reported that they lived between 9 and 10 km from the nearest health facility, and the majority of caregivers reported walking to the health facility in both surveys. It took caregivers approximately two hours, on average, to reach the nearest health facility at both baseline and endline, regardless of whether they walked or traveled by other means.

### iCCM care-seeking and treatment coverage

The household survey analysis resulted in a mix of positive and negative trends (**Tables 2 to 4**). Caregiver awareness of the presence of the iCCM-trained HSA in their community was high at both baseline (90%) and endline (83%) but showed a downward trend over the project period (*P* < 0.05) (**Table 2**). Of those aware of an iCCM-trained HSA in their community, only one-third knew the HSA’s role (ie, could name two or more curative services that the HSA performs) at both baseline and endine. In addition, perceptions of HSAs as trusted health care providers (82% at baseline and 70% at endline) who are convenient sources of care (60% at baseline and 47% at endline) and providers of quality services (68% at baseline and 58% at endline) all decreased over the project period (*P* < 0.01). Among caregivers who sought care from an HSA for at least one illness, there was no change in the percentage who found the HSA at first visit (approximately 85% at both baseline and endline). Seeking care from an appropriate health care provider remained consistent over the project period (66% at baseline and 70% at endline), but the percentage of children taken to an HSA increased from 26% at baseline to 33% at endline among all cases of illness and from 30% at baseline to 41% at endline among cases of illness for which any care was sought.

**Table 2:** Household survey results: caregiver knowledge, perceptions, and care-seeking

Indicator	Baseline % (95% CI)	Endline % (95% CI)	Baseline N	Endline N	*P*-value
**Caregiver knowledge of iCCM HSA in their community**					
% aware of iCCM HSA	90.0 (83.3-94.2)	83.4 (74.7-89.6)	720	783	**0.036**
% know the role of the iCCM HSA*	35.0 (29.6-40.9)	34.0 (28.7-39.7)	648	653	0.793
**Caregiver perceptions of iCCM services**					
% view HSAs as trusted health care providers*	82.3 (77.5-86.2)	70.3 (62.8-76.8)	648	653	**<0.001**
% believe HSAs provide quality services*	68.4 (63.7-72.7)	57.6 (52.3-62.7)	648	653	**<0.001**
% cite HSAs as a convenient source of treatment*	59.6 (52.5-66.3)	47.3 (39.9-54.8)	648	653	**0.005**
% found HSA at first visit	86.5 (79.3-91.5)	84.0 (78.2-88.4)	230	312	0.541
**Sick child care-seeking†**					
% sought care from an appropriate provider	65.6 (60.7-70.1)	70.0 (65.4-74.2)	1260	1447	0.074
% sought care from HSA as first source, all sick child cases	25.7 (20.2-32.1)	33.4 (27.1-40.3)	1260	1447	**0.028**
% sought care from HSA as first source, sought any care	30.1 (23.7-37.4)	40.5 (33.2-48.2)	1076	1194	**0.012**

HSA – health surveillance assistant, iCCM – integrated community case management

*Asked only of caregivers who states that there was an iCCM-trained HSA in their community.

†Denominator for these indicators is sick child cases, not caregivers.

**Table 3:** Household survey results: sick child assessment

Indicator	Baseline % (95% CI)	Endline % (95% CI)	Baseline N	Endline N	*P*-value
**Sick child assessment, all illness cases**					
% febrile children tested for malaria	35.6 (30.0-41.7)	59.0 (53.7-64.2)	455	471	**<0.001**
% febrile children who received the malaria test result	96.9 (92.6-98.7)	97.3 (95.0-98.6)	162	337	0.761
% children with cough and difficult or fast breathing who were assessed for fast breathing	25.6 (20.6-31.4)	38.5 (33.5-43.7)	441	489	**<0.001**
**Sick child assessment by HSA at village clinic, sought care from HSA**					
% febrile children tested for malaria	0	61.7 (52.7-70.0)	126	196	**<0.001**
% febrile children who received the malaria test result	0*	98.4 (93.3-99.6)	0	121	–
% children with cough and difficult or fast breathing who were assessed for fast breathing	29.6 (21.0-40.0)	55.8 (46.0-65.1)	98	147	**<0.001**

CI – confidence interval, HSA – health surveillance assistant

*There were no cases or number of cases was too small to calculate a percentage.

**Table 4:** Household survey results: Sick child treatment

Indicator	Baseline % (95% CI)	Endline % (95% CI)	Baseline N	Endline N	*P*-value
**Sick child treatment, all illness cases**					
% confirmed malaria cases that received ACT*	84.4 (77.0-89.8)	92.4 (87.9-95.4)	122	238	**0.032**
% confirmed malaria cases that received ACT promptly*,†	57.4 (47.5-66.7)	59.2 (52.5-65.7)	122	238	0.747
% diarrhea cases that received both ORS and zinc	18.4 (13.8-24.1)	21.2 (16.9-26.3)	364	387	0.398
% diarrhea cases that received ORS	70.1 (64.4-75.1)	68.5 (63.2-73.3)	364	387	0.664
% diarrhea cases that received zinc	21.4 (16.8-26.9)	24.0 (19.1-29.8)	364	387	0.439
**Sick child treatment by HSA, all illness cases**					
% confirmed malaria cases that received ACT*	0.0	34.9 (25.6-45.4)	122	238	**<0.001**
% confirmed malaria cases that received ACT promptly*,†	0.0	25.2 (17.8-34.4)	122	238	**<0.001**
% diarrhea cases that received both ORS and zinc	7.1 (4.3-11.6)	10.6 (7.2-15.4)	364	387	0.183
% diarrhea cases that received ORS	27.8 (21.5-35.1)	30.2 (23.6-37.8)	364	387	0.533
% diarrhea cases that received zinc	8.2 (5.1-13.0)	11.1 (7.7-15.9)	364	387	0.263
**Sick child treatment by HSA, sought care from HSA**					
% fever cases that received ACT*	59.7 (51.2-67.7)	54.4 (45.2-63.2)	124	184	0.446
% fever cases that received ACT promptly*,†	52.4 (43.9-60.8)	38.6 (30.6-47.3)	124	184	**0.026**
% confirmed malaria cases that received ACT*	0‡	89.0 (79.1-94.6)	0	91	–
% confirmed malaria cases that received ACT promptly*,†	0‡	64.8 (53.8-74.5)	0	91	–
% diarrhea cases that received both ORS and zinc	22.2 (14.1-33.3)	27.4 (20.0-36.3)	117	146	0.421
% diarrhea cases that received ORS	82.9 (72.8-89.8)	76.7 (68.5-83.3)	117	146	0.320
% diarrhea cases that received zinc	24.8 (16.2-36.0)	28.8 (21.3-37.6)	117	146	0.525

CI – confidence interval, ACT – artemisinin-based combination therapy, ORS – oral rehydration solution, HSA – health surveillance assistant

* Includes only children 5-59 months old, per national malaria treatment protocol.

† “Promptly” indicates the same day or next day after the onset of fever.

‡There were no cases or number of cases was too small to calculate a percentage.

Sick child assessment indicators showed improvement over the project period (**Table 3**). The percentage of febrile children who had blood drawn for malaria testing increased overall from 36% at baseline to 59% at endline, and among cases for which care was sought from an HSA, the percentage increased from 0% at baseline to 62% at endine (*P* < 0.001). At baseline, HSAs were providing presumptive malaria treatment; mRDTs were introduced during RAcE. The percentage of children with cough and difficult or fast breathing who had their respiratory rate assessed for fast breathing increased overall from 26% at baseline to 39% at endline, and from 30% at baseline to 56% at endline among cases for which care was sought from an HSA (*P* < 0.001).

The overall percentage of febrile children with confirmed malaria who received artemisinin-based combination therapy (ACT) treatment increased from 84% at baseline to 92% at endline (*P* < 0.05), but the percentage who received ACT treatment the same or next day following the onset of fever (prompt ACT treatment) was slightly less than 60% at baseline and did not change over the project period (**Table 4**). The percentage of confirmed malaria cases treated by an HSA increased from 0% at baseline to 35% at endline (*P* < 0.001), and the percentage of confirmed malaria cases treated promptly by an HSA increased from from 0% at baseline to 25% at endline (*P* < 0.001). Among those who sought care from an HSA, the percentage of confirmed malaria cases treated by an HSA was 89% at endline, and the percentage treated by an HSA promptly was 65%. The percentage of febrile children who received ACT treatment from an HSA, among those who sought care from an HSA remained between 50% and 60% over the project period, but the percentage that received ACT treatment promptly decreased from 52% to 39% over the same period (*P* < 0.05).

Although care-seeking from an appropriate health care provider for children with diarrhea was 64% at baseline and 70% at endline, the percentage of children with diarrhea who received both oral rehydration solution (ORS) and zinc, as specified in the national treatment guidelines, was only 18% at baseline and 21% at endline, and the findings were similar among those who sought care from an HSA: 22% at baseline and 27% at endline.

### Implementation strength

Forty-seven of 60 survey clusters (78%) had iCCM-trained HSAs available for interview. The remaining 13 clusters either had an HSA in the community who was not trained in iCCM, or the HSA who had been providing iCCM services in the community had passed away or had been transferred and not replaced. Among those interviewed, the median age was 36 years (range: 29-59 years), and 30% were female. The majority (62%) had completed form four; 32% had completed form two; and 6% had only a primary school education. Four of five (83%) reported residing in their catchment area, and of those who did not, 63% lived less than 30 minutes from their village clinic. It took 21% of HSAs two or more hours to travel from their village clinic to their supervisory health facility; 43% between one hour to just under two hours; and 36% less than one hour. Seventy-five percent of HSAs usually traveled this distance by bicycle, 19% walked and 6% used a motorbike.

Almost 90% of the HSAs interviewed met the Malawi MOH’s definition of a functional HSA (**Table 5**). Only 26%, however, resided in their catchment area, provided iCCM services during the month preceding the survey, and operated their village clinic for at least five days per week. HSAs, on average, managed 43.5 sick child cases during the month preceding the survey. Gaps in availability of essential iCCM medicines and supplies were common; 60% of HSAs interviewed had all essential iCCM medicines and diagnostics in stock on the day of the survey, and 36% reported stockouts of essential iCCM supplies lasting 7 or more days in the month preceding the survey. In the three months preceding the survey, two-thirds of HSAs reported being supervised and just over half reported receiving clinical mentorship.

**Table 5:** Implementation strength (N = 47 HSAs)

Indicator	Endline
**HSA residency and functionality (%, 95% CI)**	
% HSAs living in catchment area	83.0% (68.8-91.5%)
% functional HSAs, according to MOH criteria*	89.4% (76.2-95.7%)
% functional HSAs, according to stricter criteria†	25.5% (14.8-40.4%)
**HSA activity levels**	
# days HSAs report operating village clinic per week	Mean: 3.3 (±2.2)
	Median: 2.0 (range: 0-7)
# days HSAs report working from health facility in past month	Mean: 5.5 (±5.4)
	Median: 4.0 (range: 0-31)
# sick child cases HSAs treated in the past month	Mean: 43.5 (±38.9)
	Median: 37.0 (range: 0-220)
**Medicine and diagnostics availability (%, 95% CI)**	
% HSAs with all essential iCCM supplies‡ and functional timer in stock on day of survey	59.6% (44.6%-73.0%)
LA (1×6) (1 packet)	87.2% (73.7*-*94.3%)
LA (2×6) (1 packet)	78.7% (64.2*-*88.4%)
mRDT (2 tests)	89.4% (76.2*-*95.7%)
Amoxicillin (1 blister pack)	78.7% (64.2*-*88.4%)
ORS (3 sachets)	78.7% (64.2*-*88.4%)
Paracetamol (6 tablets)	89.4% (76.2*-*95.7%)
Zinc (10 tablets)	83.0% (68.9*-*91.5%)
Eye antibiotic ointment (1 tube)	68.1% (53.0*-*80.1%)
Timer (1 functional)	87.2% (73.7*-*94.3%)
% HSAs reporting no stockouts of essential iCCM supplies‡ lasting 7 or more days in past month	63.8% (48.8*-*76.6%)
**Supervision**	
% HSAs supervised, including register review, during the past 3 months	66.0% (50.9*-*78.4%)
% HSAs mentored, including clinical observation, during the past 3 months	55.3% (40.5*-*69.2%)

MOH – Ministry of Health, CI – confidence interval, LA – lumenfantrine artesunate, ORS – oral rehydration solution, mRDT – malaria rapid diagnostic test, HSA – health surveillance assistant

*(1) have provided iCCM services in past month, and (2) report operating village clinic for at least two days per week

†(1) reside in their catchment area, (2) have provided iCCM services in past month, (3) report operating village clinic for at least five days per week.

‡LA, mRDTs, amoxicillin, ORS, and zinc.

The survey data showed that caregivers were more likely to have sought care from an HSA during the two weeks preceding the survey if the HSA in their community had ACTs in stock on the day of the survey (52%) compared to HSAs who did not have ACTs in stock (16%) (*P* < 0.001). We did not find similar relationships between care-seeking and any other iCCM medicines or supplies.

Twenty percent of clusters were classified as having high access to iCCM; 50% moderate access, and 30% limited or no access. We found significant differences between clusters with limited or no access to iCCM and those with moderate or high access (**Table 6**). Differences did not exist, however, between clusters with moderate access and high access. For example, only one quarter of caregivers in clusters with limited or no access to iCCM viewed HSAs as convenient sources of care, whereas more than half of caregivers in clusters with moderate or high access found HSAs to be convenient. Furthermore, only 13% of sick child cases were brought to an HSA as a first source of care in clusters with limited or no access to iCCM, but approximately 40% of sick child cases were brought to an HSA as a first source of care in clusters with moderate or high access to iCCM.

**Table 6:** Select household survey results by iCCM access

Indicator	iCCM access*			*P*-value
**Limited/none**	**Moderate**	**High**
**% (95% CI)**	**% (95% CI)**	**% (95% CI)**
**Caregiver perceptions of iCCM services**				
% know the role of the iCCM HSA†	22.9 (15.4-32.6)	36.4 (29.7-43.8)	42.8 (29.9-56.7)	**0.032**
% view HSAs as trusted health care providers†	45.8 (36.9*-*55.0)	78.4 (70.8-84.5)	82.4 (57.3-94.3)	**0.001**
% believe HSAs provide quality services†	44.7 (36.1-53.6)	61.5 (55.0-67.7)	64.9 (53.3*-*75.0)	**0.005**
% cite HSAs as a convenient source of treatment†	24.6 (16.2-35.4)	58.8 (49.0*-*68.0)	52.8 (37.6-67.5)	**<0.001**
% found HSA at first visit	81.6 (66.8-90.7)	81.8 (73.6-87.9)	90.8 (83.0-95.2)	0.169
Number of caregivers	236	386	161	
**Sick child care-seeking‡**				
% sought care from an appropriate provider	62.9 (54.5-70.6)	75.1 (68.6-80.7)	68.1 (60.5-74.9)	**0.030**
% sought care from HSA as first source, all sick child cases	13.0 (8.7-19.1)	43.5 (34.3-53.3)	38.9 (26.4-53.1)	**<0.001**
% sought care from HSA as first source, sought any care	16.2 (10.7-23.7)	51.8 (41.4-62.0)	47.9 (34.0-62.2)	**<0.001**
**Sick child treatment**				
% diarrhea and confirmed malaria cases treated correctly by any provider	34.2 (27.3-41.9)	36.3 (29.8-43.2)	36.4 (27.9-45.8)	0.905
% diarrhea and confirmed malaria cases treated correctly by an HSA	4.9 (1.3-17.2)	20.3 (14.0-28.6)	22.3 (12.5-36.6)	**0.028**
% diarrhea and confirmed malaria cases treated correctly by an HSA, sought care from HSA	27.3 (9.9-56.2)	43.1 (34.0-52.6)	47.2 (29.3-65.9)	0.421
**Number of sick child cases**	**437**	**712**	**298**	

HSA – health surveillance assistant, CI – confidence interval, iCCM – integrated community case management

*Limited/none: no HSA providing iCCM services in cluster or HSA does not meet MOH definition of functional; moderate: HSA managed at least one sick child case in past month and operates village clinic at least 2 d/week (met MOH definition of functional); high: HSA managed at least one sick child case in past month, operates village clinic at least 5 d/week, and resides in catchment area.

†Asked only of caregivers who stated that there was an iCCM-trained HSA in their community.

‡Denominator for these indicators is sick child cases, not caregivers.

## DISCUSSION

The household survey findings showed that there were population-level improvements over the project period. At endline, a greater proportion of caregivers took their sick children to HSAs for treatment; a greater proportion of children with fever were assessed for malaria and, in turn, received ACTs only if they were confirmed to have malaria; and a greater proportion of children with cough and difficult or fast breathing were assessed for high respiratory rate for their age.

Care-seeking from an appropriate provider, however, did not increase over the project period; the increase in care-seeking from HSAs was offset by a decrease in care-seeking from other health care providers. A 2013-2014 national-level evaluation of Malawi’s iCCM programme also found no change in care-seeking from an appropriate provider as iCCM was introduced in Malawi, noting (1) that care-seeking had increased substantially for malaria, pneumonia, and diarrhea during the years just prior to the introduction of iCCM, and (2) that although care-seeking from HSAs increased during the evaluation period, care-seeking from public health facilities decreased in parallel [8].

Other studies of established iCCM programmes in sub-Saharan countries found variable results related to care-seeking. In Rwanda, a study comparing national-level data from the health management information system for a period just prior to the start of iCCM implementation and a period one year later found a decrease in sick child visits to health facilities [9]. During the same period, the number of pneumonia and diarrhea treatments by CHWs increased; however, the study did not look at overall care-seeking from an appropriate provider [9]. In Uganda, an evaluation of iCCM in eight districts showed the proportion of CHW-provided sick child treatments increased while the proportion of facility-provided treatments decreased, but the total number of treatments provided by both CHWs and facilities increased [10]. A randomized control study in the Oromia region of Ethiopia found that though good quality iCCM services were available through health posts, the majority of caregivers did not use iCCM services and levels of care-seeking outside the homes remained low two years after iCCM was introduced [11,12]. Another evaluation comparing areas with iCCM and areas without iCCM in Mozambique found that most caregivers used iCCM services if they were available in their community, rather than seek care from a health facility and that overall levels of care-seeking were higher in areas with iCCM services [13]. These results indicate that factors influencing health care-seeking behaviour must be investigated while also having a thorough understanding of the local context, including community demand and need vis-ŕ-vis the design of the iCCM programme.

In Malawi, the household surveys indicated that majority of caregivers lived far – two hours on average – from the nearest health facility. Furthermore, health facility staff can see 200 to 300 patients per day in Malawi, which can mean long wait times and overburdened staff who have little time to spend with individual patients. Increased care-seeking from well-trained, well-supplied, and available HSAs, therefore, benefits not only sick children and their caregivers but also facility staff.

Findings from the household surveys that merit further investigation include the proportion of caregivers who seek care from HSAs and caregiver perceptions of HSAs. Less than half of sick child cases in iCCM-eligible areas were brought to an HSA for care at endline. Seeking care at public health facilities was just as common as seeking care from HSAs in the surveyed HTRAs. Furthermore, among cases of illness for which care was not sought from an HSA at endline, the majority of caregivers reported a preference for a provider other than an HSA. The household survey did not capture reasons for caregiver provider preferences.

Caregiver perceptions of HSAs as trusted sources of high-quality, convenient care decreased over the project period, and perceptions of HSAs a convenient sources of care at endline were particularly low (47%). Perceptions of HSAs were notably more positive in clusters with moderate or high access to iCCM services but still indicated room for improvement, particularly around providing convenient, high-quality care.

HSA availability likely played a large part in the care-seeking and HSA perception results. HSA survey results indicated that iCCM services were not regularly available in many HTRAs. At endline, iCCM-trained HSAs were not found in almost one quarter of surveyed clusters. Because of numerous HSA post vacancies, RAcE project activities could not be implemented in all targeted iCCM-eligible areas as planned, and as of March 2017, the government had suspended new HSA recruitment due to lack of funds [14]. HSA posts were vacant for a variety of reasons including promotions, transfers, retirement, and deaths. Other HSAs were usurped by health facilities to assist with their heavy workloads or became data clerks at health facilities. Some district managers did not allow HSAs who were not residents of their catchment areas to attend iCCM trainings or to provide iCCM services.

HSA residency has been a long-standing issue in Malawi’s iCCM programme. HSAs are centrally recruited and may be assigned to a district other than where they are from or where their family lives, which has been reported to negatively affect HSA retention and availability within their assigned communities [6]. HSAs who do not reside in their catchment areas often live elsewhere because suitable accommodations are not available, they have spouses who work in market towns or live with their families [6,15]. A national cellphone survey of HSAs in Malawi found that only 70% of HSAs lived in their catchment areas [4], a result similar to our findings when clusters without HSAs providing iCCM services are included. In one study about HSA selection and assignment, researchers found that limited village clinic hours, which result from the numerous other responsibilities that HSAs have, were most pronounced among communities that have non-resident HSAs [1].

HSA availability is also affected by their tremendous workload. HSAs are officially responsible for performing 262 tasks, of which iCCM-related tasks are just a small proportion [7]. This is large number of tasks in and of itself, but a recent HSA workload analysis found that HSAs were actually being assigned more than 500 tasks [7]. The analysis also showed that 42% of an HSA’s official tasks were added through task-shifting guidelines implemented in 2014 and that 21% of tasks could be shifted to others at the community level [7].

Most HSAs surveyed were functional, according to the MOH definition, but only one in four were living in their catchment area, actively providing iCCM services during the month preceding the survey and operating their village clinic at least five days a week. Therefore, across all surveyed clusters, 70% of the target population had access to an HSA providing iCCM at least 2 days per week, but only 20% had access to a resident HSA providing iCCM at least 5 days per week.

Although HSAs spend a limited amount of time operating their village clinics on a weekly basis and a sizable proportion of caregivers do not seek care from HSAs, HSAs are being accessed for treatment. On average HSAs saw more than 10 cases per week or 44 cases per month, which is similar to findings from another study conducted in Malawi [4].

Stockouts of essential iCCM supplies may have also affected caregivers’ perceptions of HSAs, and consequently, care-seeking from HSAs. Despite programme support through RAcE and supply chain support tools such as c-Stock, HSAs still experienced shortages of iCCM medicines and supplies that limited their ability to provide high-quality care. A previous study acknowledged that limited district budgets and the inability to adequately forecast also contributed to iCCM programme stockouts in Malawi [1]. RAcE field monitoring data showed that health facilities used iCCM medicines meant for village clinics, particularly amoxicillin dispersible tablets, which were not being procured by the government. Others have also reported that iCCM supplies have been used by health facility staff in Malawi [14]. Efforts to identify and address supply chain management system issues at facility and HSA levels are needed.

Both the national iCCM evaluation and the RAcE HSA survey found supervision and clinical mentorship levels to be below the 80% MOH targets. Distances to HSAs’ village clinics and transportation gaps are key barriers [16]. Numerous attempts have been made to address these issues with limited sustained success, but the MOH is currently developing an integrated approach to HSA supervision so that supporting HSAs is less demanding of over-stretched facility staff and more efficient [14]. More regular, high-quality supervision and mentorship could lead to better case management, which could in turn affect caregivers’ perceptions of HSAs and care-seeking from HSAs. The quality of supervision provided to HSAs, however, is unknown and should be assessed; one study suggested that improving the quality of supervision had greater impact on quality of care than increasing only the frequency [17].

The household survey results also showed some suboptimal treatment patterns. Most notably HSA treatment of diarrhea with both ORS and zinc was low at baseline and did not improve during the project. Provision of zinc was the limiting factor in providing appropriate treatment; approximately two and a half times as many diarrhea cases received ORS compared zinc, regardless of source of care. The HSA survey showed, however, that most HSAs had zinc in stock at the time of the survey, and few reported stockouts of a week or longer in the month before the survey. The reasons for low provision of zinc by HSAs are unclear, but the trend likely went unnoticed by routine iCCM programme monitoring because the HSA monthly reporting forms do not include separate fields to indicate treatment with zinc and ORS. Further qualitative follow-up with HSAs, health facility staff, and community members is needed to understand the barriers to appropriate diarrhea treatment.

Moderate access to iCCM services was associated with increased care-seeking from HSAs, increased correct treatment of sick child cases by HSAs, and more positive perceptions of services provided by HSAs compared to areas with limited or no access. Areas with high access to iCCM did not show further improvements above areas with moderate access. These findings suggest that although HSAs are used by caregivers if they are available, as the iCCM programme is currently implemented, increasing the number of days HSAs spend in their village clinics may not lead to increased care-seeking from HSAs and treatments provided by HSAs that one might expect. This is not to say, however, that HSAs should not be more consistently available to provide iCCM services in their communities, but that this change alone may not be sufficient to improve child health outcomes. Opportunities exist to improve this and other aspects of the iCCM programme, and the MOH is working on some of these aspects.

Based on learning from the RAcE project, the MOH rolled out new HSA supervision tools and training materials and has recruited and trained additional HSA supervisors. The MOH plans to expand these efforts throughout the country and also to orient health center staff so that they can provide better supervision and support to the iCCM programme. The MOH has also developed a National Community Health Strategy[18] that aims to make community-level services more accessible and improve community awareness and demand for iCCM services and national guidelines to clarify the roles and responsibilities of HSAs and other community-level cadres who perform health-related work [7].

The MOH and its development partners could also strengthen the iCCM programme by filling more HSA posts, training and supporting more existing HSAs to provide iCCM services, ensuing iCCM supplies are more consistently available, and reducing HSAs’ workloads. The current workloads of HSAs, particularly those providing iCCM, is not appropriately aligned with their levels of compensation and support. Further, HSA workloads fluctuate in sub-national areas according to needs of donor-funded projects. The MOH should consider revising HSA job descriptions to account for evidence-based needs, effectiveness of interventions, and capacity of HSAs to deliver services effectively and with greater availability to communities.

### Limitations of the study

First, the household surveys provide estimates for the four RAcE project districts as a whole; they were not powered to provide district-specific estimates. Second, RAcE project activities were not implemented in all iCCM-eligible areas of the project districts because several HSA posts were vacant or did not have HSAs trained in iCCM and operating a village clinic. Third, the sampling frame was based on census EAs, which did not align perfectly with HTRAs, so some EAs included in the sampling frame may have included areas not eligible for iCCM, or an active iCCM-trained HSA may have been serving only a subset of an EA at the time of the endline survey. Lastly, caregiver recall of malaria diagnostic testing has been shown to be poor, which could have affected the malaria assessment and treatment indicators calculated [19].

## CONCLUSIONS

Availability of well-equipped and supported HSAs is critical to provision of iCCM services in eligible communities. Additional qualitative research is needed to examine the challenges and to inform potential solutions. Malawi’s mature iCCM programme has a strong foundation and the potential for improvement to strengthen the continuity of care from communities to facilities and to ultimately improve child health outcomes.
